# Mobility concepts and access to health care in a rural district in Germany: a mixed methods approach

**DOI:** 10.1186/s12875-018-0733-6

**Published:** 2018-05-02

**Authors:** Lisa Schröder, Kristina Flägel, Katja Goetz, Jost Steinhäuser

**Affiliations:** Institute of Family Medicine, University Hospital Schleswig-Holstein, Campus Lübeck, Ratzeburger Allee 160, 23552 Lübeck, Germany

**Keywords:** Germany, Mixed methods, Mobility, Primary health care, Rural health

## Abstract

**Background:**

Western countries are facing the challenges of an imminent shortage of physicians, especially general practitioners. As a consequence longer travel times to doctors’ practices may arise. This study aimed to investigate the mobility behavior of a rural population in terms of medical consultations.

**Methods:**

An exploratory mixed-methods design was conducted in the Waldshut district of the federal state Baden-Württemberg in Germany. Focus groups and a single telephone-interview with representatives, occupationally affiliated with mobility in the district (e.g. representatives of public transport, nursing-services or the District Office Waldshut), were performed in 2016 and analyzed using Mayring’s structuring content analysis. A questionnaire based on the collected qualitative data was subsequently distributed to a random sample of 1000 adult inhabitants living in the Waldshut district. Quantitative data were analyzed employing descriptive statistics.

**Results:**

Qualitatively, four focus groups and one single telephone-interview with a total of 20 participants were performed. Therein the necessity of reaching a nearby general practitioner and the importance of individual motor traffic was emphasized. Novel mobility modes of ride sharing and telemedicine were controversially discussed as future transport and consultation options, respectively.

Quantitatively, 277 questionnaires (27.7%) were valid and included in our analysis. Mean age was 51 years (SD = 18.5) and 58% (*n* = 160) were female. Irrespective of the mode of transport 60% (*n* = 166) expected to reach their general practitioner within 15 min. Using the possibility of multiple answers 47% (*n* = 192) stated to use a car in order to reach their general practitioner, public transport was used by 5% (*n* = 19). Nearly 80% (*n* = 220) could imagine sharing a car with well-known persons for consultations. Turning to a general practitioner via telemedicine was imaginable for 32% (*n* = 91).

**Conclusions:**

Individual motor car traffic seems to be an important factor in providing accessibility to rural medical care. As a supplementation, web based ride sharing has economic and structural potential for reaching a doctor’s practice. However, familiarity and trustworthiness need to be guaranteed within this flexible transport mode. Furthermore, telemedicine may be a future approach in order to reduce travel time to a doctor’s practice.

## Background

As in other western countries, rural areas in Germany are undergoing a transition concerning physician shortages and the age of their inhabitants. The percentage of the elderly is increasing, whereas the young are steadily moving into cities. Thus, the rural population is decreasing, aging and becoming increasingly multi-morbid [1–4].

Primary health care (PHC) is known to be of high benefit for these patients [5]. PHC is expected to provide acute and chronic medical service closely to patients’ homes and to function as a gatekeeper and coordinator. Strong PHC leads to higher and equitable health levels, lower costs and improved patients’ satisfaction [6, 7].

In Germany PHC is mostly provided by general practitioners (GP) and its importance is strengthened by the Healthcare Provision Act (VStG). GPs are the most frequently consulted physicians in Germany [8]. Without counteractions however, the locally already beginning shortage of GPs will aggravate. In Germany an undersupply of GPs is defined as a supply rate less than 75% of the relevant planning area. Within this care planning, one GP is to take care of 1671 patients [9].

The GP shortage derives from demographic change, shifted priorities of postgraduate trainees and urbanization. Demographic change impacts not only the German population but also the physicians [4, 10–12]. The number of GPs in Germany is estimated to drop from around 59.000 by 7000 until 2020 [13, 14]. Additionally newly qualified GPs prefer working part-time, in group practices and increasingly as employed physicians. Therefore it is expected that three newly qualified GPs need to follow two experienced ones into practice in order to ensure general medical care in its current state. Currently as few as 10% of all postgraduate trainees choose to become a GP. Accordingly, the GP shortage will increase remarkably [15]. Hence especially rural areas might experience a decline in close to home medical service.

Baden-Württemberg (BW) is a German federal state with a population of 10.88 million. It is located in south-west Germany, close to Switzerland and France [16]. Demographically, 35% of BW’s 7102 GPs are older than 60 years as of 2016 [17]. These 1127 GPs are aged between 65 and 92 years. In a former study BW’s mayors had declared that practices already had to shut down due to doctors’ shortage [18]. Additionally, an existing sense of undersupply was documented [19].

BW is divided into 35 districts of which nine are defined as rural based on the definition of the Federal Institute for Research on Building, Urban Affairs and Spatial Development (BBSR). According to the BBSR rural areas are defined by a population density below 150 inhabitants per km^2^ [20]. One of these districts is Waldshut, located far in the south-west of BW bordering on Switzerland. Its countryside is dominated by the mountains and valleys of the Black Forest offering transportation opportunities in a north-south axis and along the river Rhine. The local public infrastructure of especially the River Rhine’s hinterland however seems to be deficient [21].

The Waldshut district is divided into seven towns and 25 municipalities, whereof seven municipalities have got more than 7000 inhabitants. With its 145.2 inhabitants/km^2^ Waldshut belongs to the least densely populated districts in BW (average population density in BW: 305 inhabitants/km^2^; average population density in Germany: 227 inhabitants/km^2^) [20, 21].

Demographic change continues in Waldshut: To date there are 95 GPs working in Waldshut and 43% of these doctors are older than 60 years. Therefore they are remarkably older than the standard in BW [17]. As described above they will face problems concerning their replacement [4, 12, 22].

In regard to the demographic change and the shortage of GPs not only health service but also accessibility of local practices come to the fore. The behavior of Germany’s rural inhabitants in terms of mobility and consultations however is unknown. By using the district of Waldshut this study aimed to explore inhabitants’ mobile needs and necessities in the context of rural medical services to possibly develop alternative mobility concepts.

## Methods

A sequential exploratory mixed methods research design had been chosen for this study. Qualitative focus groups and a single interview were followed by a quantitative survey built upon the results of the qualitative data. Triangulation has been used as a result-oriented validation strategy: For this purpose the explorative phenomena was viewed from different perspectives aiming for an in depth and extensive analysis. Along with differentiation of results triangulation aims to converge the qualitative and the quantitative results [23].

### Focus groups and single interview

For the purpose of enabling an open discussion, focus groups were formed. As one instrument of qualitative research they enable to share experience and knowledge, to explore correlations and to discuss on a common ground [24, 25]. Since one participant was not able to attend the focus groups, that person was interviewed one-on-one via telephone. All focus groups as well as the single interview were semi-standardized and moderated by a researcher with medical background (JS: Professor for Family Medicine and director of the Institute of Family Medicine, University Hospital Schleswig-Holstein, Campus Lübeck; KF: postgraduate physician and researcher at the Institute of Family Medicine, University Hospital Schleswig-Holstein, Campus Lübeck). The purpose of the qualitative design was to get a subjective in-depth opinion from Waldshut’s inhabitants. The interview guide was created based on literature research with reference to mobility and rural health [4, 26–28]. This work presents results of the following four questions:

1. How do you define ideal access to medical service?
2. How do inhabitants of your district get to a doctor’s office if necessary?
3. How do you think about combined transport, so that already existing logistics (such as pharmacies, labs or parcel services) combine their services for future mobility of patients?
4. What do you think of ride sharing which you can book and pay online?

The discussants and interviewee had to be adult representatives of one of the following three different professions affiliated with mobility in Waldshut: mobility in the district (i.e. representatives of public transport and voluntary transport), mobility in health care (i.e. representatives of medical-, community- and nursing-services) or administration and mobility (i.e. representatives of the District Office Waldshut). In recruiting these participants we were supported by the district office of Waldshut.

The focus groups and interview participants (*n* = 40) were contacted via email or telephone. Before starting with the focus groups and the single interview all participants were fully informed about the study both verbally and in writing. A corresponding consent form was signed by all participants. In addition everyone was given a short-form socio-demographic questionnaire.

### Survey

A survey questionnaire was developed based on the responses from the qualitative interviews. For this work relevant survey questions pertaining to ideal visiting hours, investment of travel time to medical services, current and future mobility concepts, barriers of using public transport as well as socio-demographic characteristics were compiled. Likert scaling was used for future mobility concepts. Nominal scaling was used for all other items.

After piloting the questionnaire using think aloud techniques with two persons [29], which resulted in very minor linguistic changes, it was distributed via post to a representative random sample of 1000 adults living in the Waldshut district. The random sample was retrieved based on the data of the Municipal Information Processing Organization Baden-Franconia (KIVBF, www.kivbf.de) using following inclusion criteria: aged 18 and older, at least 20% older than 65 years, 51% female. Residents’ address data from the KIVBF were retrievable for 30 out of 32 towns and municipalities.

### Data analysis

All focus groups and the single interview were recorded digitally, pseudonymized and transcribed verbatim. Three researchers (LS, KF and JS) analyzed the transcripts according to Mayring’s structuring qualitative content analysis*.* Firstly deductive categories were inferred from the interview guide line followed by inductive categories inferred from the transcripts. LS analyzed all the material completely, whereas JS and KF split their coding. All categories and prototypical quotations were discussed and adapted in the process of analyses by the three researchers in order to generate a consistent coding agenda [30, 31]. During that process JS acted as a supervisor and mediator. After the consistent coding agenda had been finalized the coding was repeated by LS aiming to reassure completeness.

Descriptive analysis of quantitative data was performed using SPSS 24.0 (SPSS Inc., IBM). Continuous data was summarized using means and standard deviations. Categorical data was presented as frequency counts and percentage.

## Results

### Characteristics of our study samples: Qualitative and quantitative

Four focus groups with three to six participants and one single telephone interview had been conducted. The average interview duration of the focus groups was 57.8 min (SD = 6.7; min: 50 min, max: 64.3 min). The single telephone interview lasted 21 min. Mean age of the interview participants was 50.9 years (SD = 10.5).

A total of 298 surveys was returned (response rate: 29.8%), whereof 277 (27.7%) were valid and analyzed. 160 (57.8%) were female. Mean age was 51.2 years (SD = 18.5).

Table 1 shows socio-demographic characteristics of the 20 qualitative participants and 277 survey participants.

**Table 1:** Sociodemographic characteristics of the qualitative and quantitative study sample

Characteristic		Qualitative sample	Quantitative sample
		(*n* = 20)	(*n* = 277)
Age in years, mean		50.9 (SD = 10.5) (min: 24, max: 76)	51.2 (SD = 18.5) (min: 18, max: 95)
Gender, % (n)			
	Male	30 (6)	42 (117)
	Female	70 (14)	58 (160)
Origin, % (n)			
	Rural area	55 (11)	n.a.
	City	45 (9)	n.a.
Place of residence, % (n)			
	Rural area	60 (12)	n.a.
	City	40 (8)	n.a.
Occupational background, % (n)			
	Mobility in the district^b^	35 (7)	n.a.
	Mobility in health care^c^	40 (8)	n.a.
	Administration and mobility^d^	25 (5)	n.a.
Area of employment, % (n)^a^			
	Rural area	30 (6)	n.a.
	City	50 (10)	n.a.
	Rural area and city	15 (3)	n.a.

*Min* minimum, *max* maximum, *SD* standard deviation, *n.a.* not available

^a^n varies due to missing data

^b^Representatives of public transport and voluntary transport

^c^Representatives of medical-, community- and nursing-services

^d^Representatives of the District Office Waldshut

### Qualitative results

In the qualitative interviews three main topics were discussed. They contained ideal access to medical care, current mobility concepts and future mobility. The qualitative results, including categories and quotations, are presented below using the three main topics as subheadings:

### Ideal access to medical care

Three main categories were defined. These were accessibility, barriers and GP-shortage.

The general ease or challenge of reaching a doctor’s practice, hospital or pharmacy was covered. Time of journey and location were the main focus. The above named facilities should be as close as possible, i.e. in the village of residence.

"Optimally, without question, a doctor is on site, a doctor is in the village" (Participant (P) 3).

"I envision reaching my GP and the most important specialists nearby or maybe in my community" (P4).

Some interview participants claimed that opening hours and appointments were deficient, meaning that it was sometimes not possible to get an appointment promptly, especially for specialists. On Wednesday and Friday afternoons as on weekends and during summer holidays, there would often be no GP available which led to an overcrowding of hospitals and remaining GP practices.

"Well if I consider GPs or specialists, actually getting an appointment is very difficult. And then to express a wish concerning the time of an appointment is basically impossible" (P2).

"A big problem last summer was that many doctors’ practices were closed at the same time. There was no agreement concerning the timing of holidays" (P17).

Several participants experienced a GP-shortage in Waldshut which consequently weakens the ideal access to PHC. As a reason they stated that in particular rural areas are not that attractive for young doctors and their families. Additionally, it was believed that because of their adjacency to Switzerland some doctors preferred to rather work in the neighboring country due to assumed better working conditions.

“One point is that rural doctors increasingly disappear since it is not attractive for the young” (P8).

“In addition doctors may prefer to go to Switzerland because they can earn as twice as much there and are less stressed” (P16).

All of this allegedly would lead to full local practices and long waiting lists, so that some patients would rather get treatment in neighboring districts or a hospital. As a countermeasure, some participants suggested strengthening the reputation of GPs by reinforcing the importance of PHC at medical school. Others suggested creating stimuli and financial incentives for the settlement of young physicians. In order to ensure future health service quite a few interviewees proposed to set up central health centers, which combine the service of GPs, different specialists and pharmacies and which can easily be reached.

“Via obligation and pressure you will not get young physicians to work in rural regions, but instead via incentives” (P1).

“Well, I think the trend is moving more and more towards central health centers. The advantage is that public transport can react and hence stop at these centers, whereas physicians in single practices cannot be reached” (P2).

### Current mobility concepts

One main category was identified and defined as current mobility concepts.

The different transport options to a doctor’s office were listed. Participants mainly stated planning to use a car until old age. The support of family members and neighbors in terms of driving was expected and emphasized.

“The majority however will drive to a doctor’s practice with their own car as long as possible” (P4).

“Well, I think family help must not be underestimated so family or relatives are recruited for driving” (P7).

In terms of neighborly help the participants mentioned that an association of neighbors in some communities can arrange transportation and company to a doctor’s practice. Public transport was usually seen as a minor transport option: Due to infrequent bus service, deficient barrier-free access and lack of information some patients are hindered to use public transport. Furthermore the coordination of bus stop and practice location, schedules and appointments posed a challenge.

“We also have got an association of neighbors in our municipality for three, four years now. You can basically rent someone and it costs 5 € per hour. They do your shopping, drive you to a doctors’ practice and pick you up again” (P3).

“It is not that common that citizens are using bus or train” (P19).

Voluntary bus transport was mentioned as future possibility to get to a doctor’s practice. Since that option was only available twice a week and in two communities an extension of that service was claimed. Furthermore, a so called hitch-hiking bench was brought up as an interesting but inefficient mobility option. The importance of home visits by doctors for immobile patients was underlined. However, the interviewees realized the structural and economic challenges involved.

“And some individual doctors still do home visits, only a few, but not generally. It is not attractive either timewise or financially” (P10).

“There is a bench, which says “hitch-hiking bench” above it, and anyone who wants to be driven can sit on it and whoever passes (the bench) stops, (asks) where do you want to go, and then the other one gets a lift” (P16).

### Future mobility

Two main categories were found. These were future mobility concepts and telemedicine.

The possibility and feasibility of different future and potential mobility concepts for the Waldshut district were discussed. In this context combined transport refers to a dual or alternating transportation of goods and patients in delivery vehicles of parcel services, pharmacies or laboratories for instance. Combined transport however was mainly refused as trust, legal aspects and logistics caused concerns. The participants did not feel comfortable to share a car with strangers. In addition, combining transportation of patients and goods would cause insuperable legal problems. In regard to logistics, infrastructure and equipment for theses possible services may not be practical.

“(Combined transport) would fail because many would be scared to get into the vehicle of strangers as a lonely passenger” (P3).

“No, well no, I can see a clear difference between transport of passengers and goods” (P16).

On the other hand mobility concepts of web based ride and cost sharing were regarded with favor. This implies sharing a car journey with other travelers for a fee. Even though the same as the above mentioned concerns occurred, the participants could imagine sharing a vehicle with friends and neighbors. In particular, economic and structural advantages of that driving mode were emphasized. Hence, efficiency and utilization of cars in the district would be maximized.

“Clearly it would be much more economical to arrange car sharing on a platform” (P1).

“Actually, no additional (car) journeys would be needed since they already all exist” (P2).

Furthermore, telemedicine was addressed by the interviewers and turned out to be a controversial subject. Providing an initial assessment of medical conditions via video consultation could be especially beneficial if it leads to a reduced need of travelling to a GP’s practice. Moreover, the participants could imagine using telemedicine for consultations between patients, GPs (or qualified personnel from the practice) and specialists aiming to gather expert opinions. In addition, this would save travel time too. However, these digital solutions could only be successfully implemented if patients were examined and treated by an analog doctor frequently as well. Many qualitative participants countered that telemedicine would weaken the physician-patient-relationship. Consequently, an entirely web based consultation was strictly refused.

“In my opinion it can only be the first step of medical care, which leads in the end to a consultation in a doctor’s practice” (P20).

“One can surely handle minor diseases without going to a doctor’s practice, such as a cold or something like that” (P3).

Since all potential future mobility concepts are somehow affiliated with the internet, it was in all focus groups and the single interview concerned that the elderly might have restricted access to these novel options. Nearly all participants stated that the older citizens would probably not be able to handle web based mobility. For the young generation however it would pose a viable option.

“And I think internet access is always difficult for the elderly” (P14).

“I can envision (telemedicine) playing a role in the future because the number of people who can use the internet is increasing” (P6).

### Quantitative results

The quantitative results are subsequently presented on the basis of the qualitative results using the same three main topics as subheadings:

### Definition of ideal access to medical care

Reaching a GP within 15 min regardless of transport mode expected 60% (*n* = 166). In order to see a specialist more minutes were tolerated: 37% (*n* = 103) would invest 16–30 min and 41% (*n* = 113) 31–60 min. Almost 50% (*n* = 137) wanted to be within 16 to 31 min to a hospital. More than three quarters (*n* = 213) of the participants expected to reach a pharmacy within 15 min. Further details are displayed in Table 2.

**Table 2:** Disposed investment of travel time to medical services in one direction

Medical persons and services, % (n)^a^				
	GP	Specialist	Hospital	Pharmacy
≤ 15 min	59.9% (166)	4.0% (11)	26.7% (74)	76.9% (213)
16–30 min	33.2% (92)	37.2% (103)	49.5% (137)	18.8% (52)
31–60 min	3.2% (9)	40.8% (113)	15.2% (42)	1.1% (3)
> 60 min	0.7% (2)	8.3% (23)	3.2% (9)	0.4% (1)
Don’t know	0.4% (1)	2.9% (8)	1.4% (4)	1.1% (3)

^a^n varies due to missing data

Addressing the consequences of unavailable GPs, 25% (*n* = 70) stated having received alternatively medical treatment in a hospital and 28% (*n* = 78) in a neighboring district within the past 12 months.

### Current mobility concepts

Using the possibility of multiple answers the 277 survey participants indicated using a car among other transport options 259 times (63%). Self-driving were 47% (*n* = 192). A minority was either driven by family members, neighbors or friends. Public transport use to reach a GP made up 5% (*n* = 19). In general, 98 participants stated that there are problems reaching a GP practice via public transport. 91 of them specified these barriers. With almost 40% (*n* = 57) the main problem of the latter was a lack of barrier-free access. Two survey participants admitted that they don’t know how to get information on schedules. Table 3 shows more details.

**Table 3:** Transport mode (*n* = 254) and public transport barriers of reaching a GP’s practice (*n* = 91)

Transport mode^a^	% (n)
By foot	18.3 (75)
Bicycle	9.5 (39)
Motorbike	2.0 (8)
Car (self-driving)	46.9 (192)
Car (family)	13.4 (55)
Car (friend)	1.5 (6)
Car (neighbor)	1.5 (6)
Taxi	2.2 (9)
Public transport	4.6 (19)
Public transport barriers^a^	% (n)
Barrier-free access	38.8% (57)
Coordination of appointment and schedules	30.6% (45)
Local connection of practice and bus/train stop	29.3% (43)
Information on schedules	1.4% (2)

^a^Multiple answers possible

### Future mobility concepts

In terms of combined transport the answers were rather balanced: 30% (*n* = 82) would use combined transport for a consultation, whereas 35% (*n* = 98) cannot imagine using such a delivery vehicle. The other third showed a neutral attitude or no assessment. Concerning ride sharing, more than half of the survey participants (*n* = 151) could not imagine getting into a strangers car for a lift. However, almost 80% (*n* = 217) would give familiar persons a lift or share a car with well-known persons (*n* = 220) in order to get to their doctor (Table 4). The quantitative participants were willing to pay an average of 36 Euro Cents (SD = 25.4) per kilometer.

**Table 4:** Future mobility concepts of combined transport and ride sharing

Mobility concepts, % (n)^a^					
	Combined transport	Driving a stranger	Driving a well-known person	Riding with a stranger	Riding with a well-known person
Totally agree	11.9 (33)	8.7 (24)	51.3 (142)	6.5 (18)	48.4 (134)
Agree	17.7 (49)	9.4 (26)	27.1 (75)	5.4 (15)	31.0 (86)
Neutral	13.4 (37)	17.7 (49)	6.1 (17)	13.7 (38)	7.6 (21)
Not agree	17.3 (48)	21.7 (60)	2.9 (8)	25.6 (71)	1.1 (3)
Totally not agree	18.1 (50)	25.3 (70)	0.0 (0)	28.9 (80)	1.4 (4)
Don’t know	12.3 (34)	8.3 (23)	6.9 (19)	9.0 (25)	5.4 (15)

^a^n varies due to missing data

In terms of telemedical services 32% (*n* = 91) could imagine consulting a GP via video consultation. Whereas 19% (*n* = 53) preferred using that application together with qualified personnel from the practice. Nearly one half of all participants (*n* = 125) could envision consulting a specialist together with their GP for an expert opinion. Table 5 in the end shows the quantitative results regarding telemedical services.

**Table 5:** Future concepts of telemedical applications

Telemedicine with…			
	GP^a^ % (n)	Qualified practice staff^a^% (n)	GP plus Specialist^a^% (n)
Totally agree	17.3 (48)	7.2 (20)	19.1 (53)
Agree	15.5 (43)	11.9 (33)	26.0 (72)
Neutral	18.1 (50)	15.5 (43)	11.6 (32)
Not agree	13.4 (37)	17.3 (48)	8.3 (23)
Totally not agree	24.5 (68)	31.0 (86)	20.2 (56)
Don’t know	6.9 (19)	9.7 (27)	10.5 (29)

^a^n varies due to missing data

## Discussion

The purpose of this study was to explore and determine current status and future mobility possibilities in the context of rural health care. As a main finding the necessity of having access to a nearby GP was especially emphasized. The majority was prepared to travel up to 15 min to visit a GP for non-emergency reasons. Compared to other countries the willingness to invest time is relatively low: A majority of patients living in rural Australia were willing to travel more than 30 min for access to a GP [32], although it must be taken into account that spatial distances and population density in Australia differ to Germany. Nevertheless, the German attitude towards demanding nearby GPs in rural areas needs to be reflected. In this respect article 72 of the Basic Law for the Federal Republic of Germany is noteworthy. It postulates the “establishment of equivalent living conditions throughout the federal territory” [33]. Therefore future studies could focus on the interpretation of that article in the context of health care.

In order to antagonize a potential GP shortage and its resulting mobility challenges different approaches need to be considered: Meeting the challenges of the GP shortage medical schools in particular are trying to strengthen the reputation of potential and future GPs on multiple levels. For instance 80% of Germany’s medical schools have already established Institutes for Family Medicine [34]. Moreover the Masterplan for Medical Education 2020 (“Masterplan Medizinstudium 2020”) opens the possibility of an admission quota of up to 20% for prospective rural GPs at medical school [35]**.** Focusing internationally on the medical curricula of countries similarly affected by the (imminent) GP shortage, such as the United Kingdom and the United States of America, an increased and early exposure of medical students to PHC and subsequently general practice seems to be necessary and promising when encouraging medical students to pursue a career as a GP [36, 37].

Proceeding to the German postgraduate training several federal states, including BW, have established an educational network program for trainees in family medicine. The so called “Verbundweiterbildung plus” aims to offer structured training and to strengthen the identity and network of (rural) GPs [38]. Moreover a nationwide survey with trainees in family medicine revealed a general willingness to work in a rural general practice: Meeting their identified influencing factors “family friendly surrounding, the rural village itself and cooperation with colleagues”, 77% could envision going rural. Furthermore, a positive tendency towards working as an employee in group practices can be observed [39]. In this respect studies performed in the United Kingdom and Switzerland add international accordance concerning the positive career path general practice can offer in terms of employment, working atmosphere and work-life balance [40, 41].

The daily work of rural GPs on the other hand may be optimized by a certain task delegation: A study performed in the rural county Oberspreewald-Lausitz, Germany proved a positive effect for GPs in terms of reduced working hours and improved number of patients by delegating home visits to trained practice staff [42]. Hence all these restructurings and upward rural trends may be able to counteract the imminent GP shortage.

With regard to the derived mobility challenges in both the qualitative and quantitative study the importance of using a car for health care mobility was emphasized, whereas public transport was rated as a minor transport option. Our findings concur with former national studies showing that people living in rural areas mainly use their (own) car in order to ensure their mobility [4, 43]. Our study adds accordance for medical consultations.

Moreover, the acceptance of flexible and alternative mobility options was investigated among the rural population: In the context of medical consultations web based ride sharing was prioritized over combined transport. This former transport mode is known to increase the mobility level if the demand of certain users is very specific, similar or low. In the context of medical care this can apply to a transport of patients, whose destination is always the same single practice, central health center or hospital [44]. Since underage and elderly persons were identified as having major restrictions concerning rural mobility web based ride sharing could be a possible solution [45]. For this purpose the assumed substandard internet access of the elderly needs reflection. In Denmark for instance more than 80% of the 65 to 74 year old and more than 50% of the above 75 aged are using the internet frequently [46]. Hence web based mobility concepts of ride sharing may have a high potential for the future and in particular for the internet-oriented as well as aging generation. In contrast, combined transport, especially due to the legal barrier of the German passenger transport act and a lack of personal trust, is currently not eligible for the district.

With regard to the mentioned challenges of PHC on weekends and during summer holidays a mobile clinic operating in rural areas could possibly bridge temporary medical gaps. In Germany mobile clinic concepts, as a supplement to rural primary health care, are in a developmental state; therefore final results are rare [47, 48].

Looking further into the digital future telemedicine and video consultation might be an approach to medical care in rural areas. This mode of telemedical care however is hardly established in Germany [49, 50]. Hence its acceptance and feasibility, especially with Germany’s rural population, needs further investigation. Furthermore a combination and integration of telemedicine and home visits carried out by trained and delegated staff may be a possible solution in the context of medical care of rural immobile and increasingly multi morbid patients. In this regard a study performed in Pomerania, Germany showed that this type of delegation is a feasible possibility when approaching the imminent GP shortage [50, 51].

Immerging even further into revolutionary mobility concepts, autonomous vehicles may provide an opportunity for rural healthcare and possibly more freedom for immobile patients. So far, data of implementing that novel driving mode within the healthcare sector have not been obtained. Therefore the feasibility and possible impact of driverless cars for rural consultations need further investigations.

### Strengths and limitations

Qualitative research does not aim to generalize data but rather to explore new fields of research. In this context an observer bias regarding generating and analyzing qualitative data cannot be fully eliminated. For reduction of the bias, the medical background of the moderators was indicated to all participants and open questions without empathetic commentary were used at all times. Moreover, the intersubjective conformability, as a key quality criteria of qualitative research, was provided by documenting all steps of the research process, analyzing the data by three researchers as well as using qualitative coding [23].

Concerning the focus groups as one instrument of qualitative research, participants with strong and resolute attitudes may have biased the discussions with implicit arguments. Though due to the sensible conduction by our experienced moderators this bias was minimized and balanced subjective statements of all participants could have been gained. The single interview on the other hand led to an extension and consolidation of our findings.

Concerning the quantitative data it must be reflected that not all municipalities and towns of the Waldshut district were included. Nevertheless, the response rate of 30% can be considered as a typical response rate of postal questionnaires [52].

Overall this study was performed in one single district in Germany with very specific socio-demographic and geographic conditions. Hence it cannot be deduced that the results are transferable to other rural regions.

## Conclusions

In terms of providing future rural (primary) health care by guaranteeing unlimited access individual, private transport is likely to be a crucial factor in Germany. In addition, especially web based ride sharing could be a flexible and alternative mobility option for rural areas in order to reach a doctor’s practice. However, the key elements of trust and familiarity have to definitively be assured. Consequently, higher utilization of existing cars could increase the mobility of Waldshut’s rural inhabitants. Moreover, due to demographic change and imminent GP shortages in rural areas in particular, increased coordination and cooperation of PHC providers seems to be necessary. Not only availability but also accessibility of GPs and other facilities of PHC have to be provided in the future. Hence local conditions in terms of needs and necessities of inhabitants and GPs as well as infrastructural conditions need to be considered.

## Abbreviations

BBSR: German Federal Institute for Research on Building, Urban Affairs and Spatial Development
BW: Baden-Württemberg
GP: General Practitioner
KIVBF: Municipal Information Processing Organization Baden-Franconia
P: Participant
PHC: Primary Health Care
VStG: Health Care Provision Act
